# Reversible regulation of stem cell niche size associated with dietary control of Notch signalling

**DOI:** 10.1186/s12861-015-0059-8

**Published:** 2015-01-31

**Authors:** Alessandro Bonfini, Marian B Wilkin, Martin Baron

**Affiliations:** Faculty of Life Sciences, Michael Smith Building, Oxford Road, University of Manchester, Manchester, M13 9PT, UK

## Abstract

**Background:**

Stem cells can respond to environmental and physiological inputs to adaptively remodel tissues. Little is known about whether stem cell niches are similarly responsive. The *Drosophila* ovary germline stem cell (GSC) niche is a well-studied model, which is comprised of cap cells that provide anchorage and maintenance signals for GSCs to maintain oogenesis. Previous studies have shown a strong link between diet and the regulation of oogenesis, making this a useful model system in which to investigate dietary regulation of the niche and its associated stem cells.

**Results:**

We show that the *Drosophila* ovary GSC cap cell niche is a dynamic structure, which can contract and expand in fluctuating dietary conditions. Cap cells are lost when adult flies are shifted to nutrient poor diet and are restored after returning flies to nutrient-rich medium. Notch signalling in cap and escort cells is similarly reduced and restored by dietary shifts to nutrient poor and rich media. In old flies decreased Notch signalling is associated with decreased robustness of the niche to dietary changes. We demonstrated using a Notch temperature sensitive allele that removal and restoration of Notch signalling also leads to a reduction and re-expansion of the niche. Changes in niche size were not associated with apoptosis or cell division. We identified two distinct roles for Notch in the adult germarium. Notch can act in cap cells to prevent their loss while activation of Notch in the flanking escort cells results in expansion of the niche.

**Conclusions:**

We provide evidence that dietary changes alone are sufficient to alter Notch signalling and reversibly change niche size in the adult in wild type flies. We show Notch acts in different cells to maintain and re-expand the niche and propose a model in which cell fate transitions between cap cells and flanking somatic cells accounts for niche dynamics. These findings reveal an unexpected reversible plasticity of the GSC niche whose responses provide an integrated read out of the physiological status of the fly that is modulated by diet and age.

**Electronic supplementary material:**

The online version of this article (doi:10.1186/s12861-015-0059-8) contains supplementary material, which is available to authorized users.

## Background

Tissue renewal and maintenance fundamentally depend on the activity of stem cells, which provide a pluripotent source to replace lost or damaged tissue. Stem cells need to be tightly controlled through regulation of their proliferation, self-renewal and differentiation. Disruption of this regulation can lead to severe consequences, such as age-related pathologies and cancer [1-3]. This important level of control is typically exerted by the microenvironments or niches in which the stem cells reside. Cellular niches are comprised of highly specialized cells, often in defined locations within tissues, which provide anchorage to stem cells and contribute to signals that regulate stem cell maintenance, proliferation and differentiation. However, it is becoming clear that stem cells are also regulated by systemic signals that provide adaptation to the physiological status of the organism, in ways that are still not well understood. These systemic signals may for example mediate the effects of nutrition and exercise on stem cell maintenance and proliferation, with possible consequences for longevity and prolonging a healthy lifespan [4-6]. Such physiological responses of stem cells and their daughter cell lineages also underlie the large capacity for remodelling of many tissues and organs observed in different organisms. For example the large changes in size of the mammary gland during pregnancy and post lactation [7], and the reversible contraction and regrowth of the *Drosophila* intestine during starvation and refeeding experiments [8,9]. Little is known however regarding whether the niches themselves can be remodelled in response to environmental stimuli. However studies using stem cell transplantation [10] and with cancer stem cells [11,12] have suggested that niches can be more flexible and dynamic structures than previously thought.

**Figure 1:**
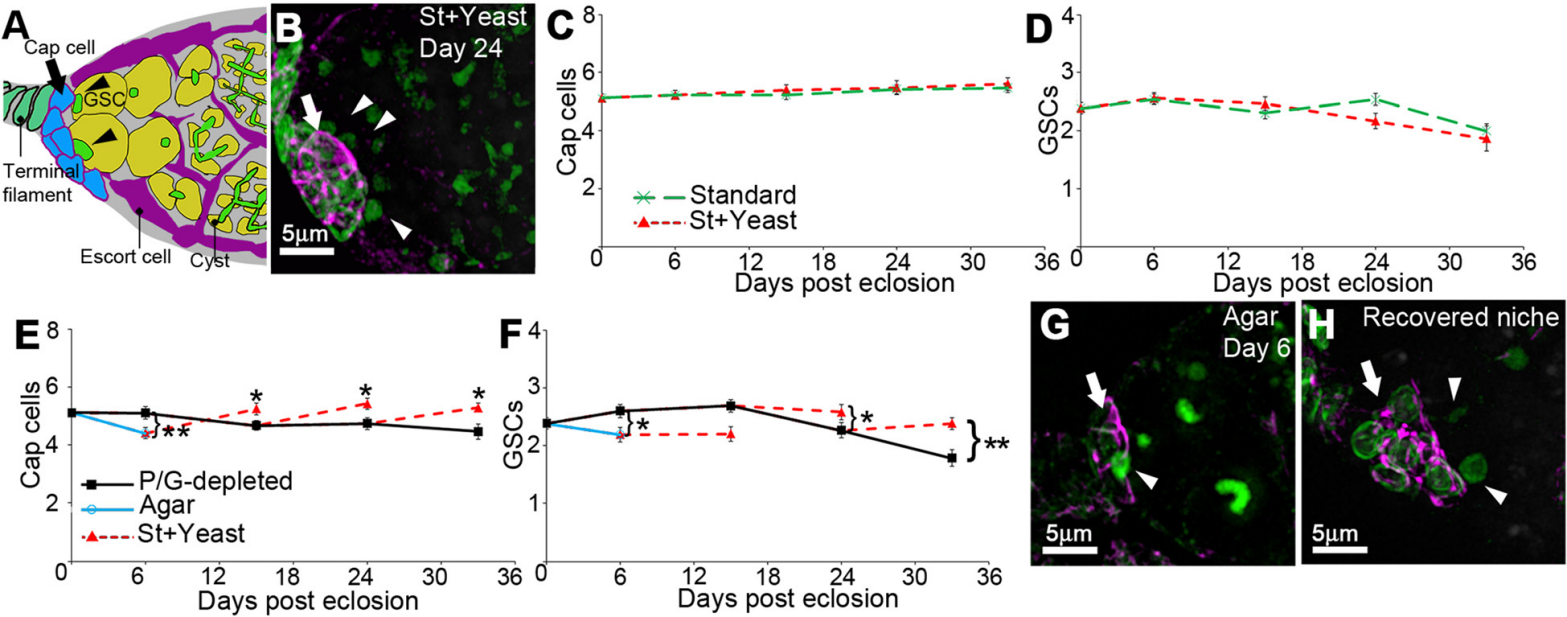
**Dietary regulation of GSC niche size.** **(A)** Diagram of anterior germarium. **(B)** Cap cell niche (arrow) marked with anti-Coracle (purple) and anti-laminC (green). GSCs (arrow heads) marked by spherical spectrosomes (anti-spectrin, also, green). **(C)** Cap cell number is maintained on standard medium or when additionally supplemented with yeast paste (St + Yeast). **(D)** Decline of GSCs with age shows no significant difference on standard food with or without yeast paste. **(E)** Cap cells decline when adults are maintained on Protein/Glucose (P/G)-depleted medium or agar only (P < 0.05, *t*-test of slope coefficients of P/G depleted or agar only compared with Standard or St + Yeast media depicted in **(A)**. Cap cells recover after shifting flies to St + Yeast, when comparing recovered time point with time of shifting to rich food. **(F)** GSCs decline similarly with age on P/G-depleted, Standard and St + Glucose, showing no significant differences. Flies kept on agar lose GSCs after 6 days compared to flies on P/G-depleted food. However GSC decline is delayed when flies initially kept on P/G-restricted food are shifted to St + Yeast, compared to either unshifted flies or to flies kept constantly on St + Yeast depicted in panel D (p < 0.05, comparing day 33 time points). Data shown in C, D and E, F as mean ± SEM and were obtained concurrently. For C, D n = 36 to 60 (except St + Yeast, day 33, n = 15); for E, F n = 32 to 60. * indicates P < 0.05, ** P < 0.01, *** P < 0.001, Mann Whitney *U*-test. **(G)** Example of reduced size niche (arrow), from fly kept 6 days on agar. **(H)** Example of recovered niche after transferring back to St + Yeast for further 9 days. GSCs marked by arrowheads. Staining as in B.

*Drosophila* oogenesis has been shown to be highly sensitive to diet. Previous work has demonstrated a sixty-fold difference in egg laying between rich and poor food conditions [13] mediated through several regulatory points during oogenesis. The germline stem cell (GSC) niche of the *Drosophila* ovary has been instrumental in establishing the niche paradigm of stem cell regulation and provides one of the most highly characterized models of niche-stem cell interactions. The ovary is composed of sub-structures called ovarioles that consist of a chain of egg chambers, each at a different developmental stage. The germ line and somatic cell lineages are derived from two stem cell populations that are located in the germarium, which lies at the anterior tip of each ovariole [14,15]. The GSCs reside in a highly defined location (Figure 1A) comprising of well characterized and distinguishable somatic cell types found at the anterior of the germarium. Five to six cap cells form the niche for two to three Germline stem cells (GSCs), providing anchorage via DE-Cadherin [16]. The cap cells in turn are located at the posterior end of the terminal filament, a line of cells extending anteriorly from the niche. The terminal filaments form during larval development and their number defines the number of niches and hence number of ovarioles in the ovary. The terminal filaments play a key role in recruiting cap cells to form the niche through activating Notch signalling in cap cell precursors [17]. GSCs locate to the niche during pupal development and begin proliferating asymmetrically to self-renew the GSC and to generate daughter cystoblasts that then undergo 4 cycles of mitosis to form a 16 cell germline cyst [18]. GSCs thus maintain egg production throughout the lifespan of the adult. Escort cells are located immediately adjacent to the niche and line the anterior germarium. Their cellular processes invade between the cysts and help propel them posteriorly [19]. Two somatic follicle stem cells (SSCs) are bilaterally located approximately midway along the germarium and give rise to follicle cells that encapsulate each cyst to form successive egg chambers. The cap cell niche provides diffusible signals such as Decapentaplegic, which regulates GSC maintenance [20,21], and Hedgehog, which acts at a distance to regulate SSC proliferation [22,23]. On a protein poor diet germline and somatic cell proliferation is reduced and cysts in region 2a/2b undergo apoptosis [13]. Insulin-like peptides and the Target of rapamycin (TOR) pathway control the response of the GSCs to nutritional status [13,24]. Insulin signalling also acts through Notch in cap cells to maintain the niche [25,26] and hence flies maintained constantly on poor diet show a loss of cap cells in aged flies compared to rich fed controls (Table S2 in [25]). Although Insulin signals are involved in long term GSC niche maintenance and rapidly affect GSC proliferation directly in response to diet, previous studies have not shown whether short-term dietary changes can have an impact on the niche size. In this study we show that the *Drosophila* ovary GSC cap cell niche can contract and expand in fluctuating dietary conditions through cell fate transitions involving flanking somatic cells. Cap cells are lost when adult flies are shifted to poor diet and Notch signalling is reduced. Returning flies to nutrient-rich medium restores Notch signalling, expands the niche and delays loss of GSCs. In old flies decreased Notch signalling is associated with decreased robustness of the niche to dietary changes. The results suggest a key role of Notch in controlling reversible niche remodelling in response to environmental change.

## Results

### Diet reversibly regulates the size of the GSC niche

To identify and score cap cells we used combined staining with anti-laminC and the cell junction marker Coracle (Figure 1A, B). The terminal filament and cap cells express nuclear membrane LaminC, which is not present in escort cells [21]. Coracle staining is enriched in cap cells compared to terminal filament and escort cells and corresponds to other common utilised markers of cap cells (Additional file 1: Figure S1) such as Zonula occludens-1 (ZO-1) [27] and Engrailed [22]. Cap cells also have a distinctive rounded morphology compared to terminal filament and escort cells. We investigated the consequence on the GSC niche of culturing wild type (WT) flies on protein rich and poor conditions by maintaining adult flies from eclosion on either our standard fly culture medium (St) or standard medium protein-enriched by supplementation with live yeast paste (St + Yeast). Addition of yeast paste has previously been shown to lead to substantial differences in rates of oogenesis and GSC proliferation [13]. However we observed no change in cap cell number over a prolonged time course (Figure 1C). GSCs showed a slow reduction in numbers over time after eclosion but this was similar in both dietary conditions (Figure 1D). Therefore we instead cultured flies on a Protein/Glucose (P/G)-depleted medium, which lacked both yeast paste and supplementary glucose. We observed a reduction in niche size that was apparent 15 days after eclosion (Figure 1E). GSC numbers declined over time in these conditions but we observed no significant difference compared to St and St + yeast media (Figure 1F compared to Figure 1D). Flies kept on agar showed a more rapid loss of cap cells which was evident by 6 days after eclosion and a significant decrease in GSCs compared to all other medium conditions (Figure 1E, F). Thus diet can affect both niche size and GSC numbers with progressively more severe and rapid effects as nutritional content is reduced.

Fly ovaries can reversibly adapt egg-laying rates to changes in diet [13]. Therefore we wondered if changes in the niche size would be similarly reversible. When flies were shifted back to St + Yeast after either 15 or 24 days on P/G-depleted medium, a recovery in cap cell number was observed (Figure 1E). We also observed higher numbers of GSCs 9 days after flies were shifted to richer nutrient conditions, compared to unshifted flies. Thus there is a delayed decline in GSC number associated with the dietary shift compared to flies kept in constant nutritional conditions (Figure 1F). A similar recovery in cap cells was observed when flies were first starved for 6 days on agar and then shifted for 9 days to St + Yeast (Figure 1E-H).

### Diet can reversibly regulate Notch signalling in the niche and escort cells

**Figure 2:**
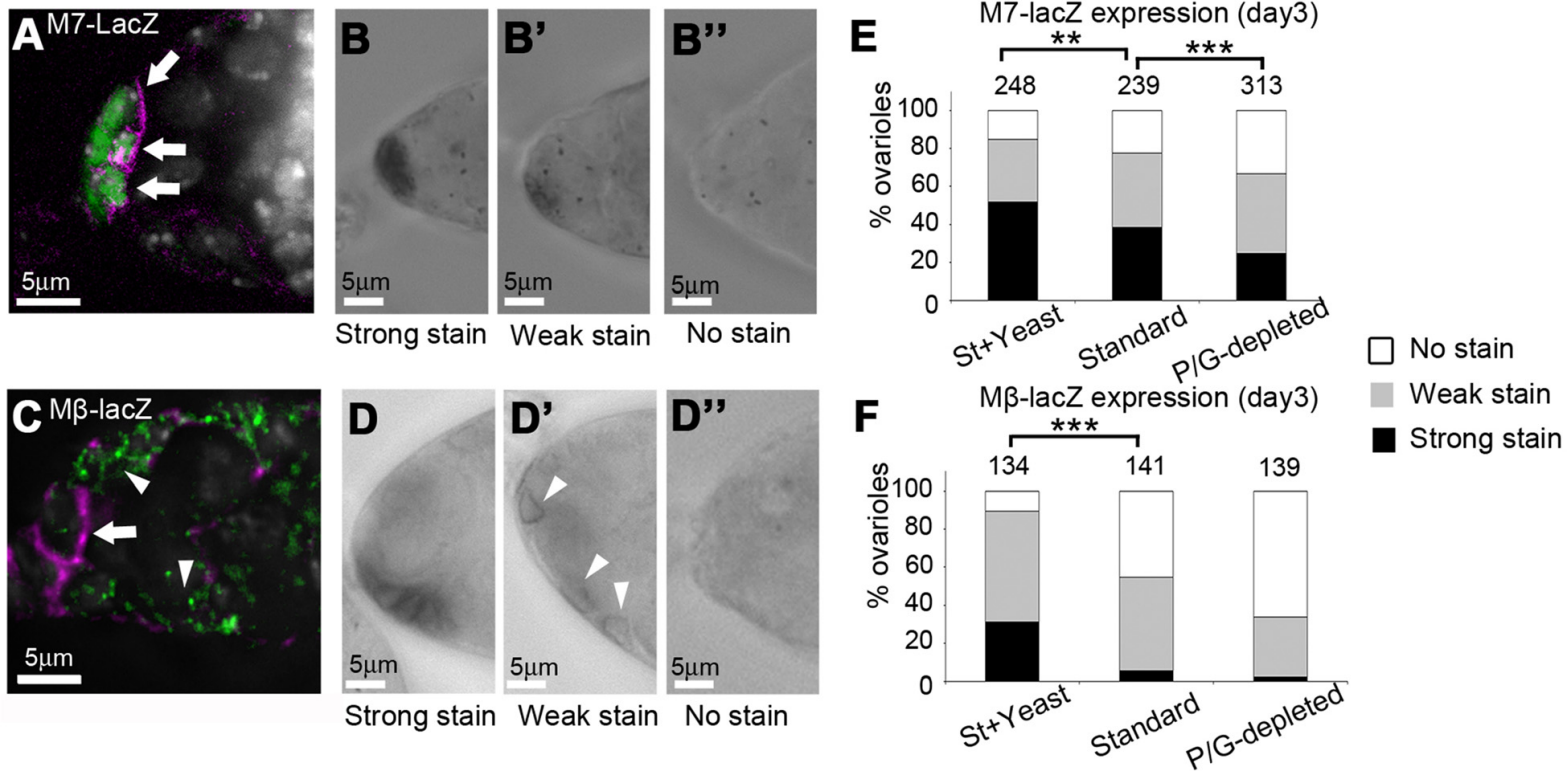
**Dietary regulation of Notch signalling.** **(A)** E (spl)m7-lacZ (M7-lacZ) expression (anti-βGal, green) in cap cells (marked with anti-Coracle, purple). **(B**-**B”)** Xgal staining of M7-lacZ germaria showing examples of strong, weak and no staining used for scoring of expression. **(C)** E (spl)mβ-lacZ (Mβ-lacZ) expression (anti-βGal, green) is stronger in escort cells (arrow heads) than in cap cells (cap cells marked with anti-Coracle, purple, arrow). **(D**-**D**”) Xgal staining of Mβ-lacZ germaria showing examples of strong, weak and no staining used for scoring of expression. Arrowheads in D’ indicate cells with weak lacZ staining. **(E)** M7-lacZ expression scored for 3 day old adults on St + Yeast, standard and P/G-depleted media. **(F)** Mβ-lacZ expression scored for 3 day old adults on St + Yeast, standard and P/G-depleted media. Data displayed as % ovarioles with strong, weak or no lacZ staining, number of ovarioles tested in each case indicated on chart. ** indicates p < 0.01; *** indicates p < 0.001, for indicated comparisons in E, F (Chi-square test).

We next investigated if different medium conditions could affect the levels of Notch signalling which is known to be required for maintenance of cap cell number in adult flies. We first examined the expression pattern of different Notch signal reporter lines since different reporters are known to be tissue-dependent [28]. As previously reported the Enhancer of Split m7-lacZ (M7-lacZ) [27,29] construct was expressed in the cap cells (Figure 2A, B) while Enhancer of Split mβ1.5-lacZ (Mβ-lacZ) [28] was found to express in the escort cells and to a lesser extent in cap cells (Figure 2C, D). M7-lacZ expression showed a small decrease in 3 day old adults on St medium compared to St + Yeast and a further reduction in signalling on P/G-depleted medium (Figure 2E). Mβ-lacZ expression showed a strong reduction on St medium compared to St + Yeast and this minimal level was not further reduced when flies were on P/G-depleted medium (Figure 2F). Thus different reporters exhibit both differences in spatial distribution of expression and different sensitivities to altered dietary composition. The stronger loss of Notch signalling on P/G-depleted medium may explain why we detected cap cell loss with this medium and not with standard medium.

**Figure 3:**
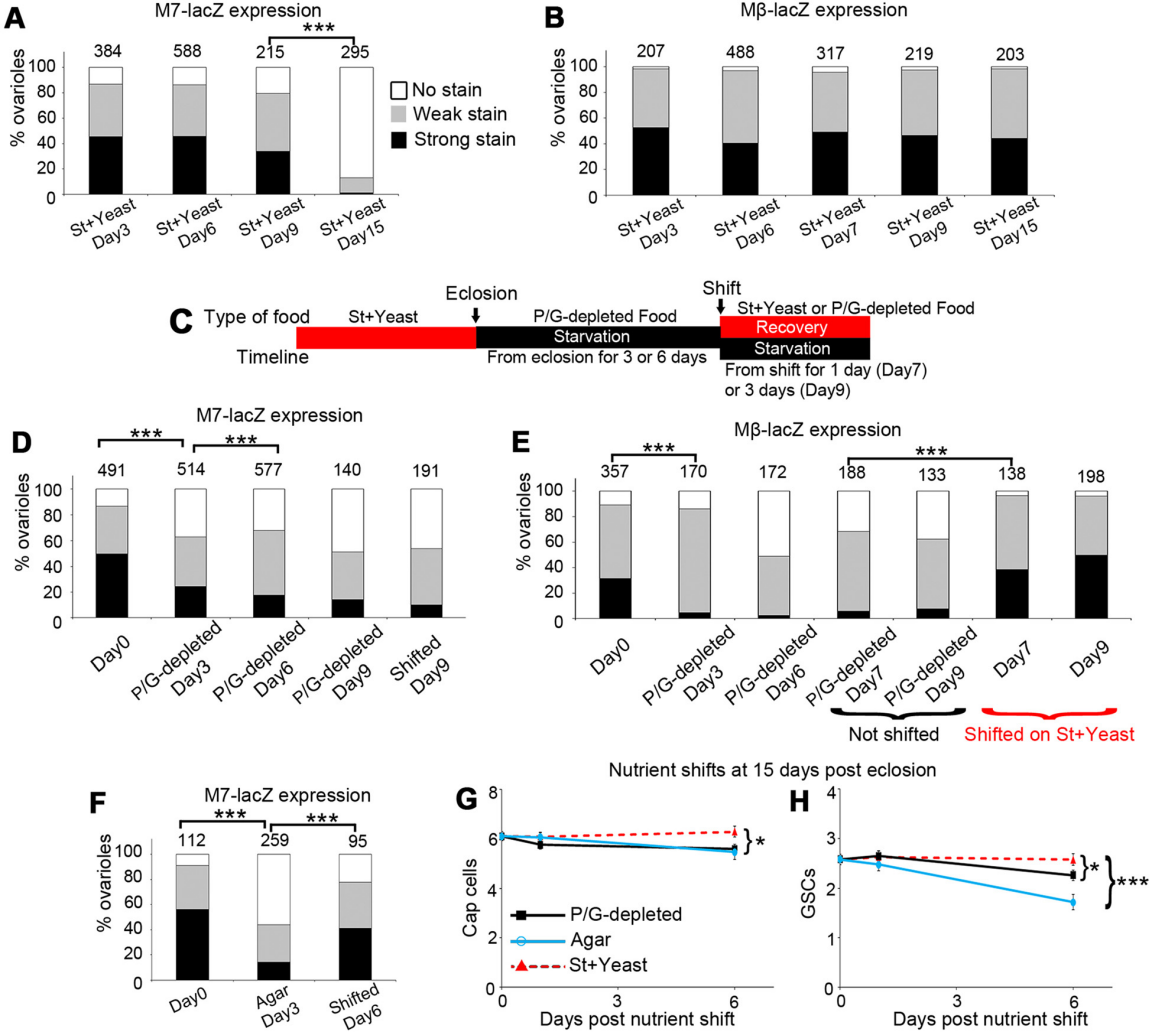
**Reversible consequences of altered dietary composition on Notch signalling.** **(A)** Time course of M7-lacZ expression in adults flies maintained post eclosion on St + Yeast. **(B)** Time course of Mβ-lacZ expression in adults flies maintained post eclosion on St + Yeast. **(C)** Time line of dietary shifts used in D, E. **(D)** M7-lacZ expression of adult flies maintained post eclosion on P/G-depleted medium or shifted to St + Yeast at 6 days post eclosion. **(E)** Time line of Mβ-lacZ expression of adult flies maintained post eclosion on P/G-depleted medium or shifted to St + Yeast at 6 days post eclosion. **(F)** M7-lacZ expression is reduced when flies are transferred to agar medium at eclosion for 3 days and restored if then shifted to St + Yeast for further 3 days. Data in A, B, D-F shown as % total ovarioles with strong, weak or no lacZ staining. Number of ovarioles examined indicated above each graph. *** p < 0.001 for indicated comparisons (Chi square test). **(G**,**H)** Adult flies were maintained for 15 days on St + Yeast before shifting to P/G-depleted medium or agar. Cap cells **(G)** and GSCs **(H)** decline in number after 6 days following nutrient downshift to both P/G-depleted and agar compared to St + Yeast controls. Data displayed as mean ± SEM * indicates p < 0.05; *** p < 0.001, Mann–Whitney *U* test, n = 21 to 60.

We investigated if Notch signalling through the different reporters changed over a time course after eclosion. On St + Yeast, M7-lacZ expression showed a small decrease at 9 days post eclosion and was strongly reduced by 15 days (Figure 3A). In contrast to this age-dependent reduction we found that the Mβ-lacZ reporter lines showed little change over time in flies maintained on St + Yeast after eclosion (Figure 3B). We next investigated how a timeline of nutrient down and upshifts (Figure 3C) affected Notch activity. On P/G-depleted medium M7-lacZ expression showed a noticeable reduction after 3 days post eclosion with further declines up to 9 days (Figure 3D). Mβ-lacZ was lost more rapidly reaching minimal expression in 3 day old adults (Figure 3E). When flies kept on P/G-depleted medium were shifted back to St + Yeast at 6 days after eclosion we found different responses of the two reporters. After shifting to St + Yeast medium, M7-lacZ expression showed no recovery (Figure 3D), but Mβ-lacZ expression levels were restored after 1 day (Figure 3E). We wondered if the age-dependent reduction of M7-lacZ expression was the underlying reason for the lack of recovery of its expression in flies undergoing nutrient up-shifts at 9 days. We therefore made use of the more rapid response and recovery allowed by keeping flies on agar-only medium. There was a strong loss of M7-lacZ expression in these younger adult flies after 3 days post eclosion in starvation conditions, followed by a recovery of M7-lacZ after a further three days on St + Yeast (Figure 3F). The reduced Notch signalling in aged flies correlated with a less robust niche, which had a shorter lag time to niche reduction and loss of GSCs after switching to a P/G-depleted medium (Figure 3G, H), compared to similar shifts of young adult flies on eclosion (Figure 1E, F).

### Loss and recovery of Notch signalling reversibly regulates cap cell number

**Figure 4:**
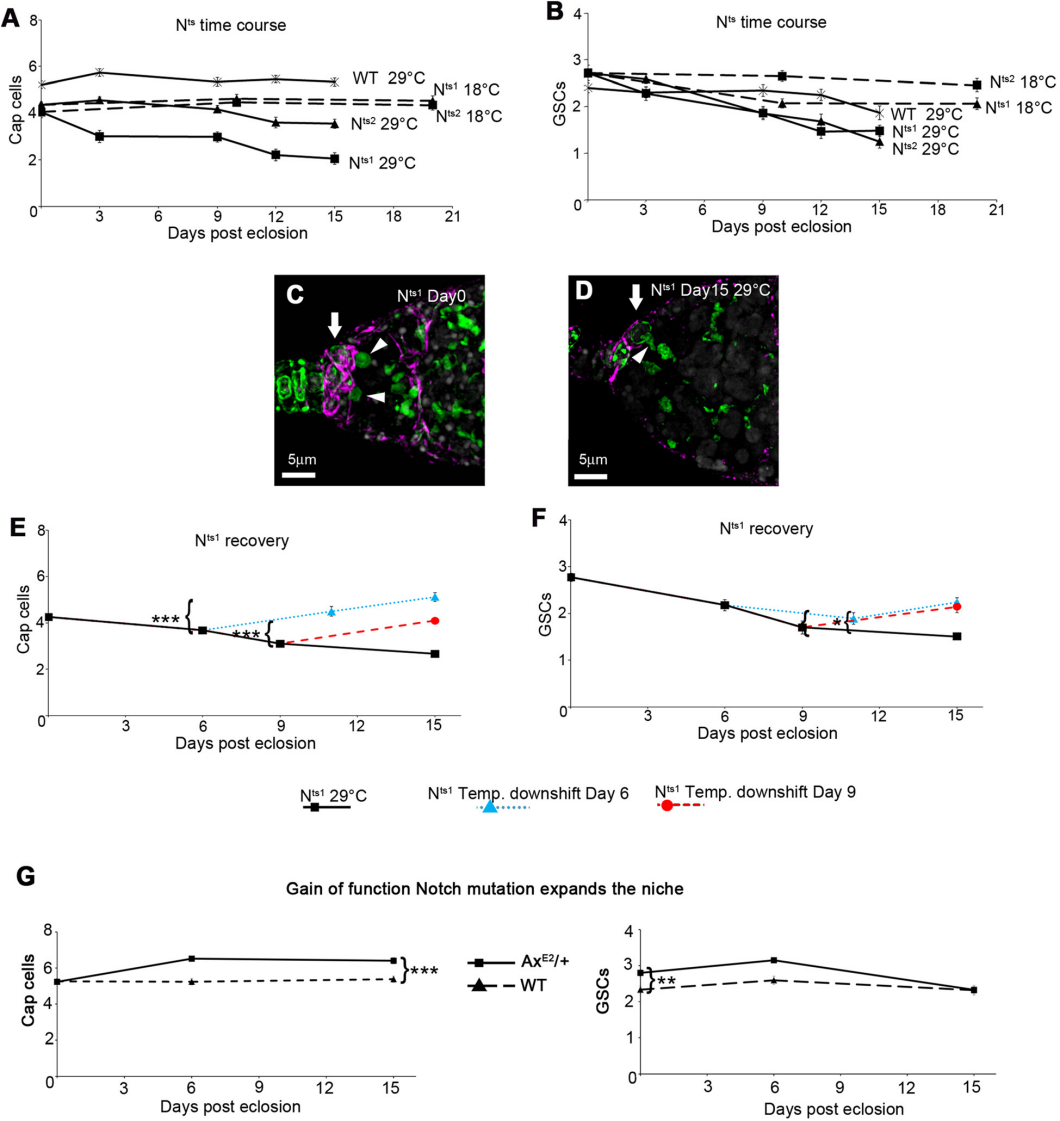
**Niche plasticity is regulated by Notch signalling.** **(A)** After development at 18°C *N* ^*ts1*^ and *N* ^*ts2*^ mutant flies have a smaller niche on eclosion compared to wild type (WT) flies but niche remains stable if flies are maintained at 18°C. Shifting *N* ^*ts1*^ and *N* ^*ts2*^ to 29°C on eclosion results in a decline in niche size while WT niche is unaffected. **(B)** GSC numbers are reduced to a greater extent when *N* ^*ts1*^ and *N* ^*ts2*^ flies are shifted to 29°C compared to similarly treated WT flies. **(C)** Cap cell niche (arrow) of *N* ^*ts1*^ germarium on eclosion stained with anti-Coracle (purple) anti-LaminC and anti-Spectrin (green). Arrowheads indicate GSCs. **(D)** *N* ^*ts1*^ mutant after 15 days post eclosion at 29°C with reduced niche size (arrow), occupied by single GSC undergoing asymmetric division (elongated spectrosome marked by arrowhead). **(E)** Decline of cap cell niche observed in *N* ^*ts1*^ flies is reversed by day 15 after a temperature downshift to 18°C at either 6 days or 9 days post eclosion compared to flies maintained at 29°C. **(F)** Decline in GSCs is similarly reversed. (A, B, E, F) Data displayed as mean ± SEM. *indicates p < 0.05; *** indicates p < 0.001, Mann Whitney *U* test for comparisons of indicated time points with adults at 15 days post eclosion. **(G)** *Ax* ^*E2*^/+ ovarioles expand cap cell numbers after eclosion compared to WT (*** indicates p < 0.001, Mann–Whitney *U* test), but GSC numbers although initially higher in *Ax* ^*E2*^/+ niches (**, p < 0.01, Mann–Whitney *U* test), eventually decline to WT levels. For (A, B) n = 29 to 62, (E, F) n = 36 to 112, (G) n = 50 to 100.

To investigate whether altering Notch signalling could also reversibly affect niche size we used the *N*^*ts1*^ and *N*^*ts2*^ temperature sensitive alleles [30], which allow normal Notch function at 18°C but produce strong Notch loss of function when flies are shifted to 29°C. Flies were shifted from 18°C to 29°C on eclosion and the population sampled at different time points for oogenesis phenotypes. As previously published [17,26] we observed a decline in cap cell number over time for both *Notch* alleles, while the niche size for WT controls at 29°C remained stable (Figure 4A). We did not find any evidence that loss of cap cells resulted from apoptosis (Additional file 2: Figure S2). *Notch* mutant flies that remained at 18°C also did not show a significant decline in cap cell number. Similarly, we observed a greater decrease in GSC number over time in temperature shifted *N*^*ts*^ mutant flies compared to WT at 29°C and 18°C controls (Figure 4B). These phenotypes were accompanied by a reduction of escort cell invasion between cysts and the onset of expected follicle packaging phenotypes (Figure 4C, D, Additional file 2: Figure S2). We investigated if the observed phenotypes could be reversed by transferring flies to 18°C part way through the time course to restore Notch activity. When the temperature down-shift was initiated 6 or 9 days into the time course we observed niche recovery. GSC numbers similarly recovered (Figure 4E, F), as did other ovariole phenotypes affecting cyst packaging (Additional file 2: Figure S2). Notch signalling thus has an impact on numerous cell types in the germarium, including the cap cells, to maintain their function in the ovary. We next investigated the effect of a dominant gain of function Notch mutant allele *Abruptex*^*E2*^ (*Ax*^*E2*^) [31]. Although, on eclosion, the niches of *Ax*^*E2*^/+ flies were comprised of a similar number of cap cells as wild type, the numbers subsequently increased in the *Ax*^*E2*^/+ niches after eclosion (Figure 4G). However GSC numbers, although they were initially higher than wild type, eventually declined to wild type levels despite the larger niche (Figure 4G), suggesting that a subtle balance of Notch activity levels in the adult germarium affects the cap cell to GSC ratio. The reversibility of the niche phenotypes shows that there are two ways that Notch can function to maintain cap cell number. Notch acts to prevent loss of cap cells but also functions to re-expand the niche.

### Spatially distinct Notch functions are associated with niche contraction and recovery

**Figure 5:**
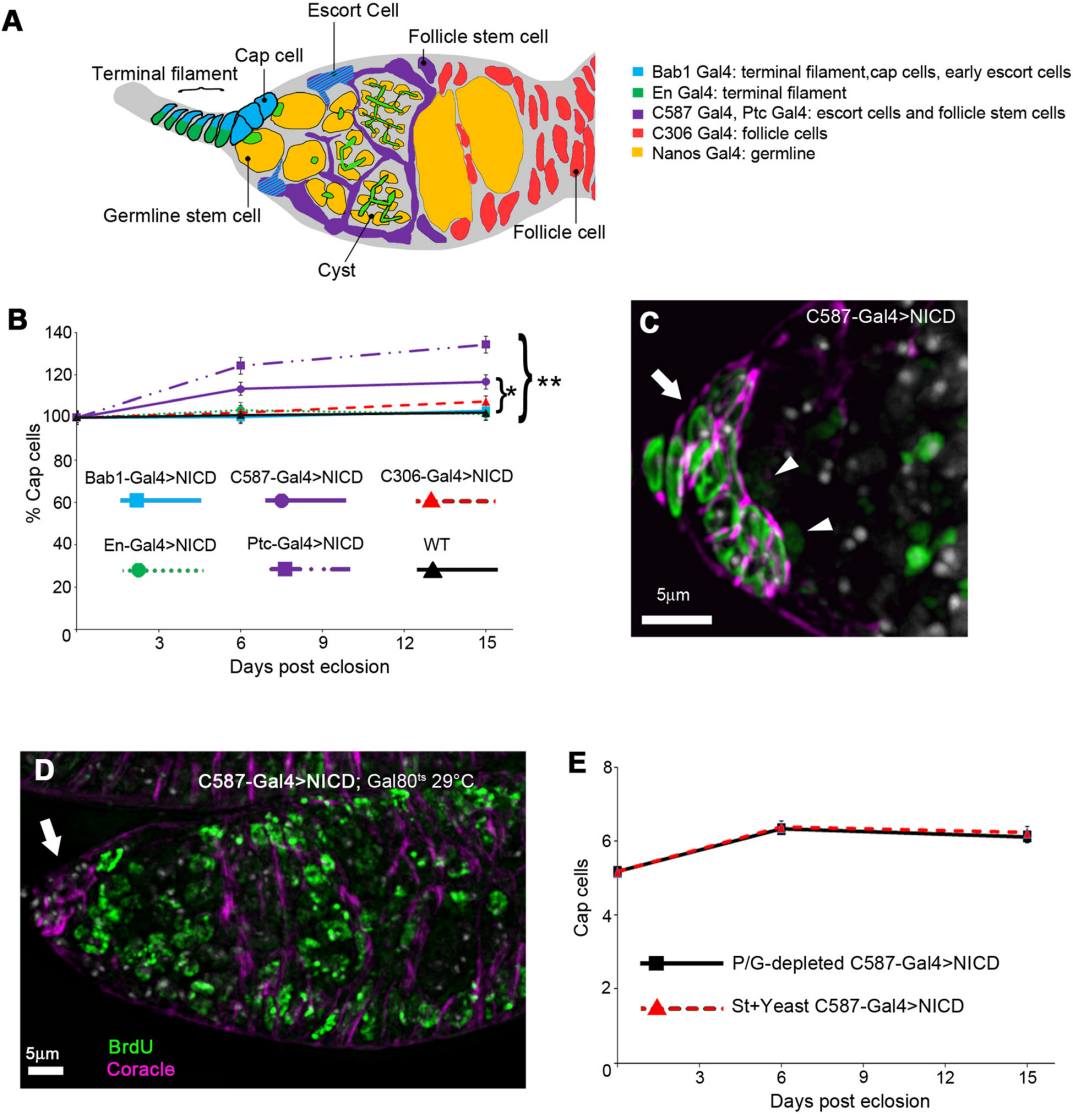
**Notch acts in escort cells to expand the cap cell niche.** **(A)** Expression patterns of Gal4 lines used. **(B)** C587-Gal4 and Ptc-Gal4 driven expression of NICD in adult niches controlled by Gal80^ts^ leads to increasing numbers of cap cells compared to WT at day 15 (respectively P < 0.05, and P < 0.001, *t*-test) while Bab1-Gal4, En-Gal4 or C306-Gal4 driven NICD expression has no effect. **(C)** Example of expanded cap cell niche (arrow) resulting from expression of NICD with C587, stained with anti-Coracle (purple), anti-LaminC (green). **(D)** Cap cells do not undergo mitosis. BrdU labelled ovariole after 15 days of expression of NICD with C587 Gal4. Cap cells (arrow), marked with anti-Coracle (purple) are not labelled with BrdU (green). Dapi is marked grey. In contrast, proliferating germ line and follicle cells did incorporate BrdU (n = 225). **(E)** C587 driven NICD expands niche similarly in flies maintained on St + Yeast and P/G-depleted media. Data in B, E shown as mean ± SEM. Data in B are normalized to % of cap cells on eclosion. (B) n = 43–61; (E) n = 59–62.

The two identified functions of Notch that control niche size may reside in the same or different cell types. Thus altered Notch signalling in different tissues might directly or indirectly contribute to either cap cell loss or niche restoration. *Notch* null cap cell clones generated during development fail to be incorporated into the niche indicating Notch function during development of the niche is cell autonomous [26]. However it is not known whether niche maintenance in the adult only depends on Notch signalling in the cap cells or whether other cell types are involved in supporting cap cell numbers. The cap cell niche is reported to be post mitotic [20] ruling out use of clone induction in adults to investigate requirements that are specific for adult functions. We therefore utilized a number of Gal4 lines to express constitutively active Notch intracellular domain (NICD) or Notch RNAi in different cell types. In order to generate Notch gain and loss of function only in the adult, we created fly stocks carrying both the Gal4 driver and a temperature sensitive Gal80^ts^ construct [32]. The bric-a-brac1-Gal4 (Bab1-Gal4) drives expression in cap cells, in the terminal filament and more weakly in the anterior escort cells [33]. In contrast Patched-Gal4 (Ptc-Gal4) and C587-Gal4 express only in adult Escort cells [34,35]. The C306-Gal4 line expresses in the follicle cell region of the germarium [36] and Engrailed-Gal4 (En-Gal4) expresses in all the cells of the terminal filament but not the cap or escort cells [37]. Nanos-Gal4 expresses in the germline but not in the somatic cells of the germarium [38]. Thus by considering the overlapping and distinctive expression patterns of different Gal4 drivers it is possible to infer the spatial requirements for Notch function (Figure 5A). Flies were kept at 18°C during development and then switched to 29°C at eclosion to induce Gal4 expression. We confirmed Gal4-Gal80^ts^ functionality by expressing UAS-CD8-GFP at 18°C and 29°C. GFP expression was observed at 29°C but not at 18°C (Additional file 3: Figure S3). To investigate if increased activation of Notch signalling can function to increase the adult niche size we expressed constitutively active NICD. Separately the UAS-NICD line and the different Gal4 drivers did not cause an increase in niche size (Additional file 3: Figure S3) while C587-Gal4 and Ptc-Gal4 driven NICD expression resulted in an increase in cap cell number (Figure 5B, C). Bromodeoxyuridine (BrdU) labelling showed that this niche expansion did not depend on cell division (Figure 5D). In contrast, no increase in cap cell number was noted with En-Gal4, C306-Gal4 or Bab1-Gal4 (Figure 5B). In the adult ovary C587-Gal4 and Ptc-Gal4 express strongly in the escort cells and not the cap cells and Bab1-Gal4 expresses in cap cells and less strongly than other drivers in escort cells. Therefore we can conclude that Notch acts in escort cells to increase niche size, presumably by a transition to a cap cell phenotype. C587-Gal4 driven NICD resulted in similar increase of cap cell numbers on both rich and P/G-depleted medium showing that nutritional control acts upstream of proteolytic activation of Notch (Figure 5E). As with *Ax*^*E2*^/+ flies (Figure 4G) the increase in cap cell number induced by deregulated Notch signalling was not associated with a net increase in GSC number (data not shown). Nevertheless we identified a population of escort cells that responded to NICD expression by increasing the numbers of niche cells expressing cap cell markers.

**Figure 6:**
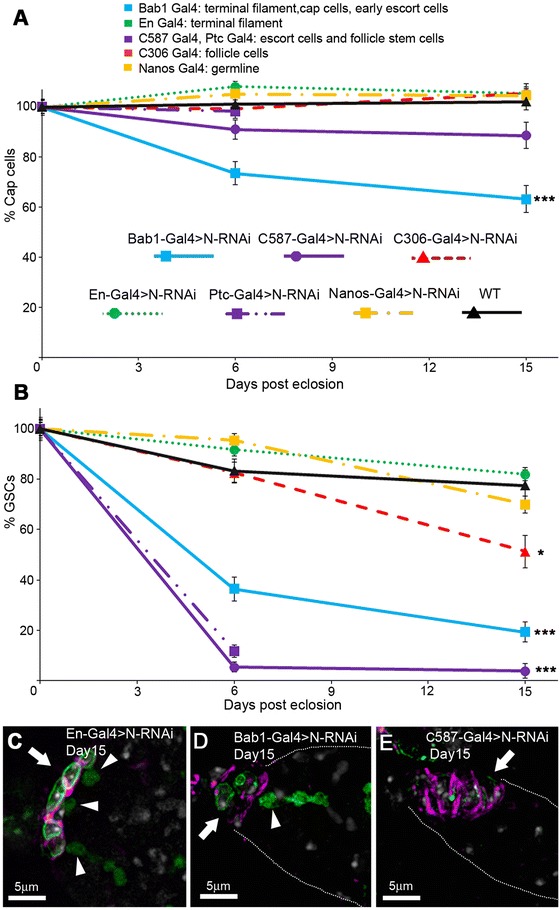
**Notch acts in cap cells to maintain adult niche.** **(A)** Adult expression of Notch RNAi was induced by temperature shift using Gal4-Gal80^ts^ system. Only Bab1-Gal4 expression produces significant decline of cap cell number in 15 day adults compared to WT (*** indicates p < 0.001, *t*-test of slope coefficients). **(B)** C587, Ptc-Gal4, Bab1-Gal4 and C306-Gal4 driven Notch RNAi cause loss of GSCs over time compared to WT (* indicates P < 0.05; *** P < 0.001, *t*-test of slope coefficients). Note flies expressing Notch RNAi with Ptc-Gal4 do not survive to 15 days. Data in B, C displayed as means ± SEM normalized to % of cap cell or GSC numbers on eclosion, n = 27–65. **(C**-**E)** Niches of 15 day old adults expressing Notch RNAi, marked with anti-Coracle (purple), anti-LaminC (green), and anti-Spectrin (also green). Arrow marks location of niche, arrowheads mark presence of GSCs. **(C)** En-Gal4 driven RNAi has no effect on niche or GSCs. **(D)** Bab1 driven RNAi reduces cap cell and GSC number, **(E)** C587-Gal4 driven RNAi reduces GSC numbers but does not affect cap cell number.

To examine which cells were sensitive to Notch loss of function we expressed Notch RNAi in adult flies, which were dissected on eclosion, and after 6 days or 15 days. The separate UAS lines were also out-crossed to WT flies. None of the lines individually showed any significant loss of cap or GSCs compared to wild type flies between eclosion and the 15 day time point (Additional file 3: Figure S3). Notch RNAi expressed by different Gal4 drivers resulted in distinct outcomes. En-Gal4 driven Notch RNAi in the terminal filament did not show any phenotypes throughout the timeline (Figure 6A-C). Bab1-Gal4 driven Notch-RNAi caused a significant loss of cap cells over time (Figure 6A, D). Reduction of GSC numbers was also observed (Figure 6B, D). C587-Gal4 and Ptc-Gal4 driven Notch RNAi showed a strong loss of GSCs and a small reduction was seen when Notch RNAi was expressed in follicle cells (Figure 6B, E). However, despite disruption to other germarium cell types, none of the drivers apart from Bab1-Gal4 resulted in a significant loss of cap cells when driving Notch RNAi expression (Figure 6A). We also observed no phenotypes when Notch RNAi was driven only in the germline with Nanos-Gal4 (Figure 6B), consistent with previous results that failed to find a function for Notch in germline cells [26,39]. By taking into account the distinctive expression patterns of the different Gal4 drivers we conclude that Notch signalling is required only in the cap cells for adult niche maintenance but indirect effects of Notch in other somatic tissues also affect GSC number. Our combined data thus shows that Notch acts separately in cap cells and escort cells to maintain or re-expand the niche.

## Discussion

Stem cells often reside in defined cellular niches adhered to specialized support cells. These niches provide a favourable environment and signals to maintain a stable population of stem cells. Changes to the activity and population level of stem cells are closely associated with tissue homeostasis, repair and remodelling to different physiological inputs. Little is known however regarding how niches are normally regulated and altered during the lifespan of an individual or how they respond to its physiological status or the organism’s environment. This is in part due to lack of a well-characterized and experimentally accessible model whereby environmental changes to the niche and to the niche regulatory signals can be observed with defined outcomes. Here we have uncovered an unexpected plasticity of the *Drosophila* ovary GSC niche. We have found that the niche can contract and re-expand in response to altered Notch signalling, a dynamic response which we have also linked to dietary regulation. We have defined two spatially distinct activities of Notch that regulate adult cap cell number. Notch can act in the adult cap cells to prevent their loss while increased Notch activation in the escort cells causes niche expansion. The reversible response of the niche to dietary composition may be an adaptive response to tune niche activity to external environmental conditions allowing a restoration of niche capacity if there is a return to conditions more productive for offspring survival. Intriguingly we found that after keeping adult flies on P/G-depleted medium, the transfer back to St + Yeast medium delayed the loss of GSCs compared to flies maintained in constant nutritional conditions whether rich or poor. It is intriguing therefore to speculate that a boost of niche signalling capacity on restoration of the niche might have a rejuvenating effect on GSC survival, but our current study does not discriminate the latter possibility from direct effects of dietary history on the GSCs. However we do note that restoration of Notch activity by temperature down shifts of *N*^*ts*^ mutants does restore the GSC capacity of the niche. Interestingly we found, using RNAi, that disrupting Notch function in escort and follicle cells separately led to reduction of GSC numbers without decreasing the niche size, while constant high level of Notch signalling in escort cells caused niche expansion without sustaining increased GSC numbers. Previously Song *et al*., using a different method to express activated Notch in mitotic clones of escort cells, also concluded that sustained high activation of Notch did not result in recruitment of additional GSCs to the escort cells [17]. Intriguingly we found that the gain of function *Ax*^*E2*^ mutation of Notch produced first a gain then a loss of GSCs in a time course analysis. This suggests the level and duration of Notch signalling is important in determining the outcome, and this has to be considered when interpreting the lack of GSC recruitment after constitutively active NICD is expressed. Our RNAi data also indicate that that multiple mechanisms involving Notch in various somatic cell types are involved in regulating the GSC to cap cell ratio in the niche.

Surprisingly our results did not replicate the finding that poor diet accelerated the decline of GSC number compared to rich diet that has been previously reported [25]. In our analysis, GSC loss was similar when flies were kept continuously on rich and poor conditions. Only starvation conditions led to a loss of GSCs a loss of GSCs compared to fed flies were less robust to later dietary shifts to poor diet. Additionally we did not observe a decline in cap cell numbers with age on rich medium or indeed on our standard medium lacking yeast paste, only when glucose supplement was removed did we observe an age-dependent decline of cap cells. It is possible that fly strain differences or different diet regimes may explain these different outcomes, but our results demonstrate that age-dependent decline of GSCs does not necessarily imply a change in cap cell number, consistent with our findings that multiple mechanisms control GSC to cap cell ratio.

The dynamic changes to the niche in response to diet correlated with changes in expression of the Notch lacZ reporters M7-lacZ and Mβ-lacZ, which showed distinct spatial and age-dependent differences. Both M7 and Mβ expression were reduced on poor diets and could be restored on shifting to rich media. But M7-lacZ also showed a decline in expression with fly age even on rich medium and hence was only restored on nutrient up-shifts in adults that were less than 1 week of age post-eclosion. The age-dependent decline of M7-lacZ expression in the cap cells was associated with a decreased robustness of the niche when aged flies were transferred to a poor diet. The plasticity and dynamic responses of the cap cell niche thus provide a read out of the physiological status of the fly, integrating both environmental and age-dependent changes. At present we do not know how the expression of the M7 and mβ reporter genes are spatially and temporally uncoupled from each other, but other studies have suggested that different co-regulatory inputs may cause tissue-dependent differences in the expression of different Notch target genes [28]. It will be interesting to discern how Notch signalling levels and responses in cap and escort cells depend on distinct or overlapping nutrient sensing mechanisms. Insulin signalling has previously been shown to play a role in maintaining a stable niche over the fly lifespan by promoting Notch activity and appears to mediate differences in cap cell number in flies maintained in constant rich or poor conditions [25,26]. Changes to Insulin signalling are therefore a strong candidate to mediate the reversible nutrient regulation of the niche size that we observe. It is likely however that additional nutrient-sensing mechanisms such as Tor signalling are also involved. For example a number of studies have shown that nutrient sensing at the cellular and systemic levels can converge to cooperatively regulate cell proliferation growth and metabolism in different physiological contexts [40-42]. Our data do not as yet rule out the possibility that the Notch response in the cap cells might reflect an indirect consequence, lying downstream of global changes initiated by nutritionally regulated signals, although the rapidity of the Mβ − lacZ response, together with previously published data indicating the cell autonomous requirement of Notch in cap cells for their maintenance [25,26], strongly argue against this caveat.

**Figure 7:**
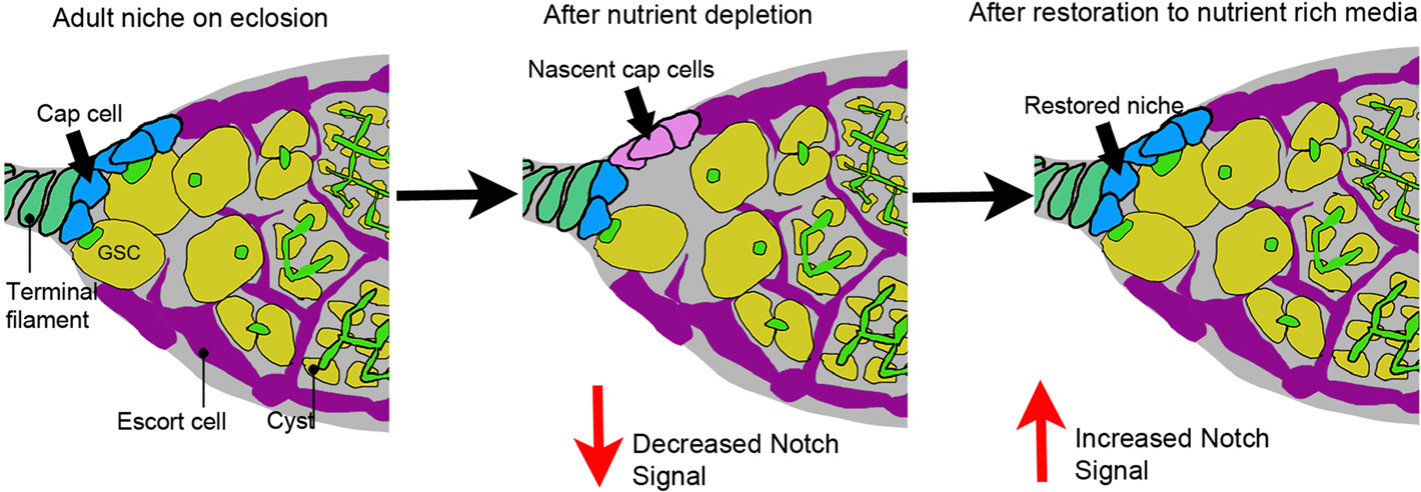
**Nutrient**-**dependent plasticity of the GSC niche.** Model of nutrient-responsive niche dynamics through Notch-dependent cell fate transitions between cap cells and an adjacent pool of nascent niche cells, likely a sub-population of escort cells, regulated by dietary inputs.

Our data ruled out the possibility of adult niche expansion through mitosis of the cap cells and instead suggests a model in which cell fate transitions between cap cells and adjacent escort cells cause the changes to adult niche size (Figure 7). Previous work has shown that overexpression of NICD in undifferentiated somatic precursor cells in the developing ovary cause greatly expanded niche sizes [17], reflecting the role of Notch in niche recruitment and demonstrating some plasticity in the developmental lineage. However in the adult ovary we found that widespread NICD expression in differentiated escort cells resulted in only a small but reproducible expansion of cap cell numbers suggesting only a limited number of adult cells retained the capacity to trans-differentiate. Interestingly a relationship has previously been shown in the *Drosophila* testis GSC niche between the niche hub cells and the adjacent cyst cells. In the male GSC niche the cyst cells are somatic cells, which surround the developing germline cysts in a way that is somewhat analogous to the escort cells and follicle cells of the *Drosophila* ovary [43]. Like the follicle cells [23] but unlike the escort cells [19], the cyst cells are continually turning over and are derived from proliferating cyst stem cells (CySCs) [43]. The hub cells are post mitotic and are developmentally derived from a common precursor to the CySCs. Interestingly in the absence of a transcriptional regulator called Lines, CySCs differentiate into Hub-like cells [44]. In the adult it has been reported that there is a steady state turnover of the Hub cell niche and that niche size is maintained by adoption of hub cell fate by CySCs [45]. However a later study found this conversion was not significant [44]. It will be interesting to determine whether this discrepancy was due to environmental differences and if similar dietary regulation affects the male GSC niche. A previous study [8] has shown that the number of GSCs and CySCs in the male testes are reversibly affected by diet but did not distinguish whether there was an outcome on the number of hub cells themselves.

Few other niches have been characterized in such detail as the *Drosophila* GSC niche, but there is some evidence that niches in other systems may have a post-developmental plasticity. For example transplanted mouse hair follicle stem cells have been shown to organize a functional niche to maintain hair growth in recipient nude mice [10]. Age-related changes to niches have also been observed. In the mouse hippocampus, for example, age-dependent quiescence of neural stem cells is associated with morphological and cytological changes to the support cells of their sub-ventricular zone (SVZ) niche [46]. Interestingly experimentally induced damage to the mouse SVZ stem cell niche is eventually repaired to produce a remodelled neural stem cell niche from SVZ progenitor cells [47]. Cancer stem cells also modify their environments to induce a supportive niche. For example myeloproliferative neoplasia remodels the bone marrow niche to favour the growth of leukemic stem cells at the expense of normal hematopoietic stem cells [48]. The induction of new cancer stem cell niche environments to support the formation of metastases has also been reported [12]. Similarly in *C.elegans*, ectopic niches for GSCs can be generated in the oviduct by mutations that lead to mis-expression of ligands for Glp1/Notch, which in *C.elegans* is activated in the GSCs [49]. These studies suggest that niches may exhibit a degree of plasticity and that some cellular environments may act as nascent niches able to be recruited in response to appropriate signals. The reversible control of niche size that we have shown in the *Drosophila* model highlights the possibility that similar niches in humans may be potential targets for therapeutic intervention.

## Conclusions

Our study demonstrates a previously unexpected reversible plasticity of the well-studied GSC niche in the *Drosophila* ovary in response to dietary nutrients, which we find associated with reversible changes in Notch signalling. We further identified different capabilities of Notch in cap and escort cells to maintain and replenish niche function and suggest gene expression changes rather than cell death and mitosis are responsible for the changes in niche size that we observe. Interestingly we showed that there are spatially distinct target gene responses of Notch activity in and around the adult niche with differential responses to dietary changes and adult age, reflected in changing robustness of the niche. Further studies that identify the mechanisms regulating Notch signalling will provide insights into how niche responses integrate these two systemic inputs.

## Methods

### Drosophila stocks and culture conditions

OregonR was used as WT control. *N*^*ts1*^, *N*^*ts2*^ alleles [30] were obtained from Bloomington Stock Centre, Indiana. Notch reporter lines E (spl)m7-lacZ [29]; E (spl)mβ1.5-lacZ [28], were gift from Sarah Bray. Transgenic expression was by the Gal4 system [50]. Gal4 lines were C587-Gal4; C306-Gal4 [51]; Patched-Gal4 (Ptc-Gal4) [52] bric-a-brac-Gal4 (Bab1-Gal4) [33]; Engrailed-Gal4 (En-Gal4) [53]. The Gal80^ts^ stock was from Bloomington, Indiana. UAS lines were Notch RNAi, Valium 20 ((Nval 20) HMS00009 TRiP stock, Harvard) obtained from Bloomington. UAS-NICD was obtained from Spyros Artavanis-Tsakonas (Harvard Medical School), and P {UAS-mCD8::GFP.L} LL6 (UAS-CD8-GFP) from Bloomington stock centre. Fly stocks were maintained at the indicated temperatures in the presence of males on standard fly medium (72 g/L maize (Doves Farm Foods), 79.3 g/L glucose (Fisher), 50 g/L autolysed yeast powder (Kerry), 8.4 g/L agar, 0.3% Proprionic acid (Sigma) and 0.27% Nipagin (Sigma) unless indicated. For protein-enriched medium (St + Yeast), standard medium was supplemented with live yeast (Sigma). Protein/Glucose (P/G)-depleted medium was as with standard recipe but without added glucose or live yeast supplementation. Starvation conditions were induced on 8.4 g/L Agar only. For BrdU labeling, flies were maintained on St + Yeast medium supplemented with live yeast paste including 5% BrdU .

### Immunofluorescence

Primary antibodies used were: guinea pig anti-Coracle, (1:5000; gift from R. Fehon, University of Chicago), mouse anti-Lamin C (LC28.26, 1:20; Developmental Studies Hybridoma Bank, DSHB, Iowa), mouse anti-alpha Spectrin (3A9 1:10; DSHB), mouse anti β-Gal (1:250; Promega), rat anti-BrdU (1:50; Abcam), rabbit anti-phospho Histone H3 (1:3000; Millipore), goat anti-GFP (1:1000; Abcam), mouse anti-FasIII (7G10 1:20; DSHB). Secondary antibodies used were: donkey anti-guinea pig RRX (1:400; Jackson laboratories), donkey anti-mouse Alexa Fluor 488 (1:1000; Invitrogen), donkey anti-mouse Cy3 (1:400; Jackson laboratories), donkey anti-rabbit Alexa Flour 488 (1:1000; Invitrogen), donkey anti-guinea pig Cy5 (1:100; Jackson laboratories), donkey anti-mouse Cy5 (1:100; Jackson laboratories), donkey anti-goat Cy3 (1:400; Jackson laboratories); donkey anti-rat RRX (1:400; Jackson laboratories).

Around 25 flies per data point were dissected. The dissected ovarioles were pooled and fixed in a medium containing 3% formaldehyde (Polyscience) in 1x PBS (Sigma) for 12 minutes washed in PBS with TritonX100 (Sigma) 0.3% (PBX) for 10 minutes and blocked with 0.5% Normal Donkey serum (Jackson laboratories) in PBX for 1 hour. The ovaries were then incubated overnight at room temperature with the primary antibody diluted in PBS, washed 5 times in PBX and a secondary antibody diluted in PBS was added overnight. Samples were then washed 5 times in PBX and mounted on microscope slides under a cover slip using Vectashield, containing DAPI (4’,6-diamidino-2-phenylindole) (Vector Laboratories). Images were captured using an Orca-ER digital camera (Hamamatsu) mounted on a M2 fluorescent microscope (Zeiss). The images were acquired as 0.5 μm Z-sections using Volocity software (Perkin Elmer) with deconvolution performed in Openlab (Perkin Elmer), and processing in Photoshop CS5 (Adobe). Images in figures displayed as 2D projections of merged deconvolved Z stacks.

### BRDU staining

Ovaries were dissected as previously described but fixed for 20 minutes. Ovaries were denatured into 3 M HCl (sigma) diluted in 0.3% PBX for 15 minutes. HCl was removed, and ovaries were incubated 3 times for 5 minutes in 0.1 M Borax (Sigma) to neutralize HCl. After this step borax solution was removed and ovaries were incubated in blocking solution with 0.5% NDS in PBX for 1 hour as previously described. Staining and mounting were performed as previously described.

### X-Gal staining

Germaria were dissected in PBS, fixed for 20 min in 1.7% glutaraldehyde (Sigma) in PBS, and then washed in PBS. Samples were stained at 37°C in 1 mM MgCl2, 6 mM K4FeIICN6, 6 mM K3FeIICN6, and 0.2% X-gal. After washing in PBS, they were then mounted in a solution containing 70% glycerol (Sigma) in PBS. E (spl)mβ-lacZ was performed on ovaries from flies heterozygous for lacZ reporter.

### Apoptosis assay

To detected apoptosis Apoptag Fluorescein Direct In situ Apoptosis detection kit (Chemicon International) was used. Dissection and stain procedures were performed as described above.

### Scoring of Cap cell and GSC numbers

Cap cells were identified by their rounded morphology, strong Coracle expression at the cell surface and presence of nuclear membrane localized LaminC stain. Because the niche is a 3D structure, for scoring cell numbers, images were acquired as a series of 0.5 μm optical sections and deconvolved using Openlab (Perkin Elmer). Individual and adjacent Z-sections could then be inspected and compared to unambiguously define the numbers of cap cells in each niche. The GSCs were identified by anti-Spectrin staining marking the spectrosome of GSCs, together with their localization adjacent to cap cells.

### Statistics

Statistics were performed with SPSS 20.0 (IBM). To analyze differences between data points two tailed Mann–Whitney *U* test was used. To compare complete time-courses, the slope coefficient was calculated through linear regression performed with SPSS20.0. Slope coefficients were then compared through independent *t* test using the following formula: t = Slope1 - Slope2/SQR [(SEslope1)^2^ + (SEslope2)^2^]. To compare X-Gal stain results, the SPSS 20.0 crosstab method with Pearson Chi Square Asymptotic 2 sided test was used.

## Additional files

Additional file 1: Figure S1. Coracle expression marks cap cells. A) Merged image showing a WT GSC niche stained with anti-Coracle (Blue), anti-Cadherin (green) and anti-ZO-1 (red). Cadherin is localised in punctate junctions in cap cells, in contrast the elevated levels of the cap cell marker ZO-1 colocalises with strong expression of the band 4.1 junction protein Coracle around the perimeter of all cap cells. B) Engrailed expression (green) marks terminal filament and cap cells. Strong Coracle expression (purple) marks the subset of engrailed expressing cells that comprise the cap cell niche. Additional file 2: Figure S2. Consequences of temperature shifts of *N* ^*ts1*^ mutant flies. A) Wild type ovariole stained for Dapi (Grey), Coracle (purple), FasIII (green) and Spectrin (green). B) Germarium of *N* ^*ts1*^ mutant ovariole after 15 days at 29 °C, stained with anti-Coracle (purple) to mark the cap cell niche (arrow) and ApopTag (green). Green staining marks an apoptotic cyst. No apoptosis was observed in cap cells of *N* ^*ts1*^ mutants at 29 °C (n = 99). C) Ovariole of *N* ^*ts1*^ mutant, after 6 days at 29°C stained with Coracle (purple), FasIII (green) and Spectrin (green), showing defective cyst packaging and incomplete separation of egg chambers. Arrow marks enlarged egg chamber containing multiple cysts. Arrowhead marks two successive egg chambers lacking intervening stalk. D) Ovariole of *N* ^*ts1*^ mutant restored to 18°C for 9 days after 6 days at 29 °C. Egg chamber formation is restored back to wild type morphology. Additional file 3: Figure S3. Cap cell and GSC scoring of Gal4 and UAS lines. (A-D) Scoring of cap cell (A, C) and GSC numbers (B, D) over time since eclosion for Gal4 lines (A, B) and UAS lines (C, D) used, after out-crossing to WT. Data shown as mean ± SEM and normalised to % of cap cell and GSC numbers on eclosion (n = 37-60). (E, F) temperature-dependent Gal4-dependent expression demonstrated using UAS-CD8-GFP driven by PtcGal4 coexpressed with Gal80ts. No GFP detected at 18°C (E) but clear expression observed at 29°C (F).

## Abbreviations

Ax: Abruptex
Bab1: Bric-a-brac1
BrdU: Bromodeoxyuridine
*C.elegans*: *Caenorhabditis elegans*
CySCs: Cyst stem cells
DHSB: Developmental Studies Hybridoma Bank, DSHB
En: Engrailed
GFP: Green fluorescent protein
GSC: Germline tem cell
m7-lacZ: M7-lacZ Enhancer of split
mβ-lacZ: Mβ-lacZ Enhancer of split
NICD: Notch intracellular domain
N: Notch
P/G-depleted: Protein/Glucose-depleted
Ptc: Patched
RNAi: RNA interference
SEM: standard error of the mean + −
SSC: Somatic follicle stem cell
St: Standard fly medium
St + yeast: Standard fly medium + yeast paste
SVZ: Sub-ventricular zone
ts: Temperature sensitive
TOR: Target of rapamycin
UAS: Upstream activator sequence
wild: Type (WT)
ZO-1: Zonula occludens-1
