# Supplementary figures and images for: Reversible regulation of stem cell niche size associated with dietary control of Notch signalling
Source: BMC Dev Biol. 2015 Jan 31;15:8. doi: 10.1186/s12861-015-0059-8 (PMC4320563; doi:10.1186/s12861-015-0059-8)

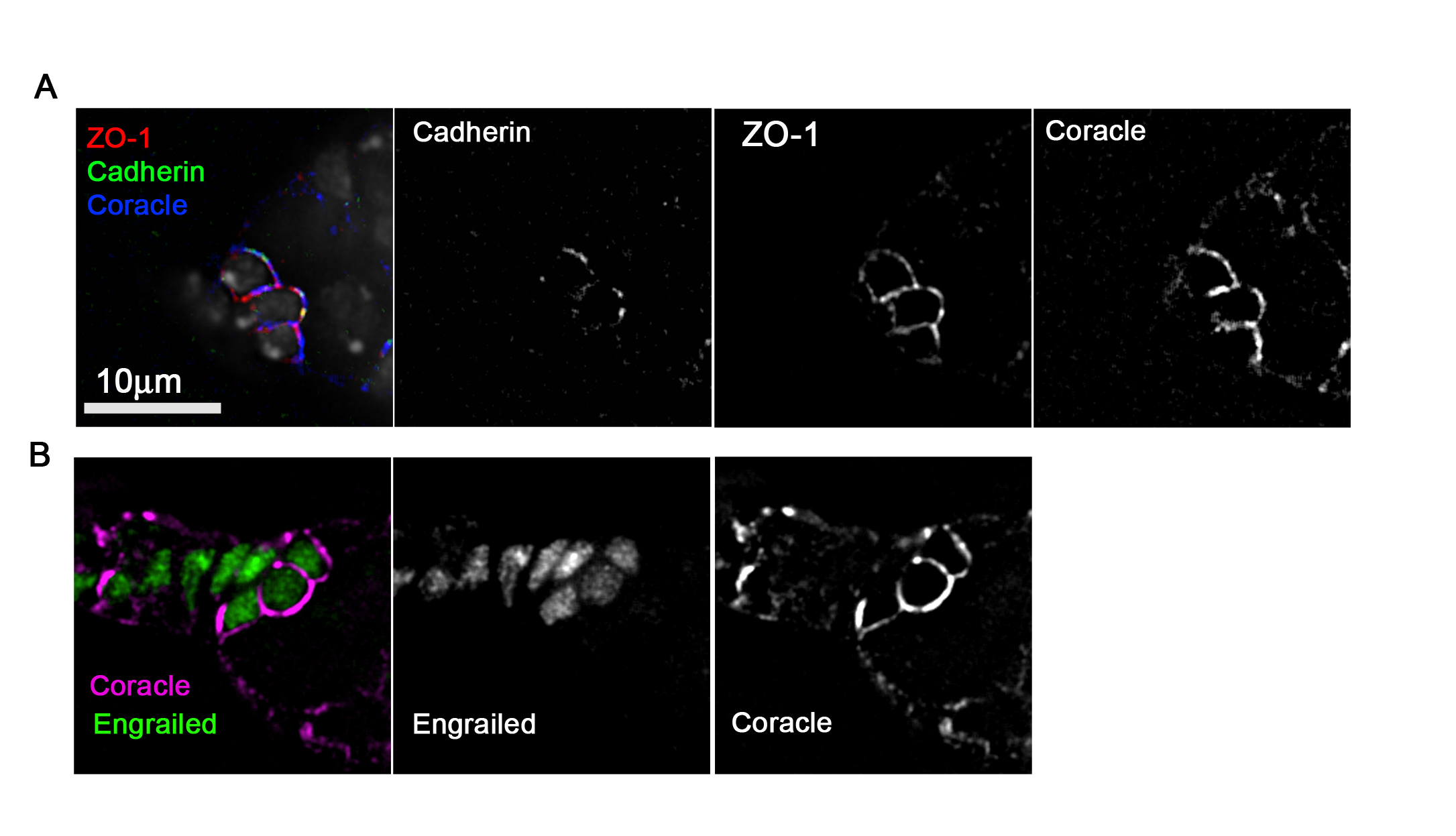

Supplement: Additional file 1: Figure S1. — Coracle expression marks cap cells. A) Merged image showing a WT GSC niche stained with anti-Coracle (Blue), anti-Cadherin (green) and anti-ZO-1 (red). Cadherin is localised in punctate junctions in cap cells, in contrast the elevated levels of the cap cell marker ZO-1 colocalises with strong expression of the band 4.1 junction protein Coracle around the perimeter of all cap cells. B) Engrailed expression (green) marks terminal filament and cap cells. Strong Coracle expression (purple) marks the subset of engrailed expressing cells that comprise the cap cell niche. [file 12861_2015_59_MOESM1_ESM.jpeg]

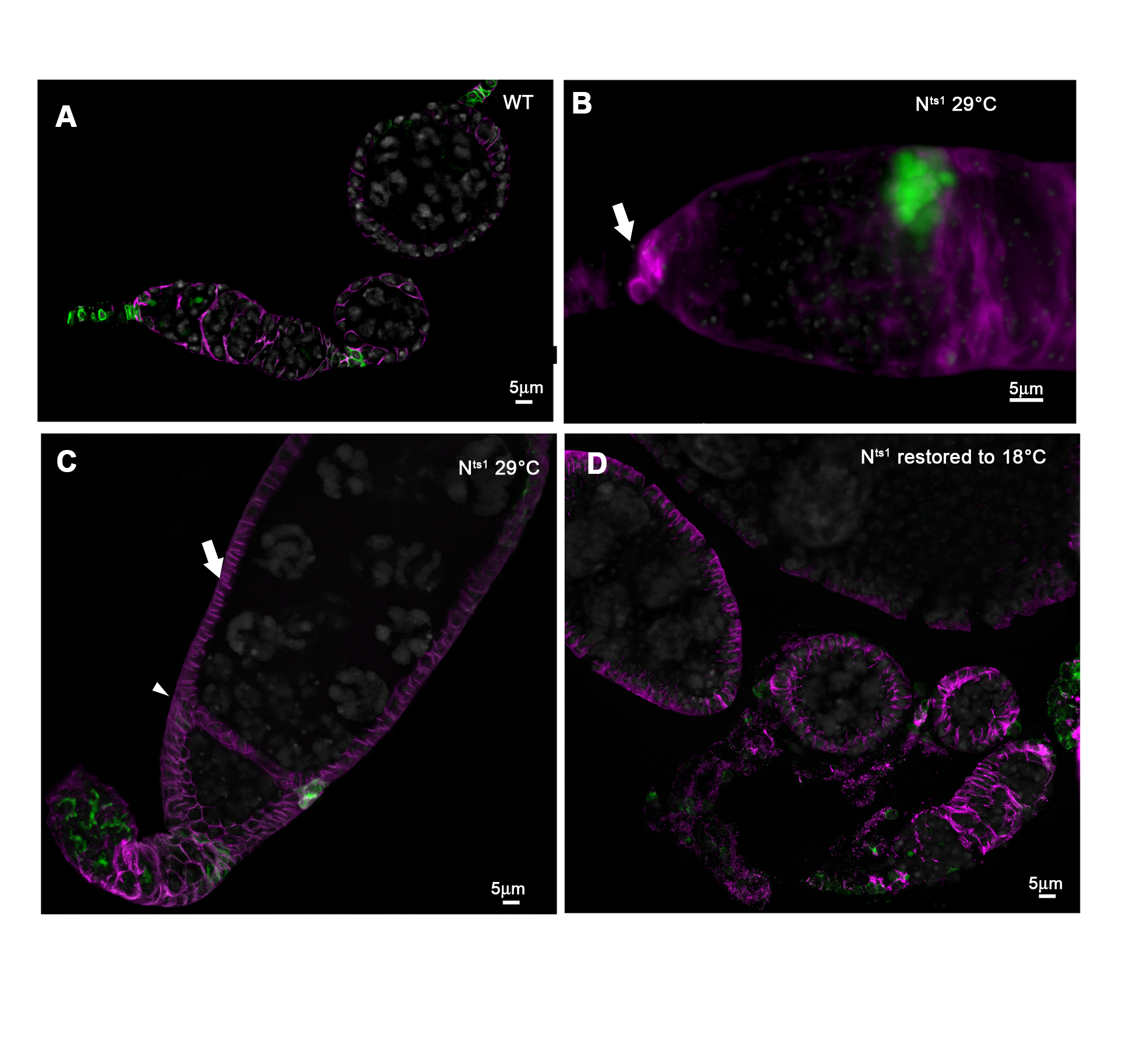

Supplement: Additional file 2: Figure S2. — Consequences of temperature shifts of N ts1 mutant flies. A) Wild type ovariole stained for Dapi (Grey), Coracle (purple), FasIII (green) and Spectrin (green). B) Germarium of N ts1 mutant ovariole after 15 days at 29 °C, stained with anti-Coracle (purple) to mark the cap cell niche (arrow) and ApopTag (green). Green staining marks an apoptotic cyst. No apoptosis was observed in cap cells of N ts1 mutants at 29 °C (n = 99). C) Ovariole of N ts1 mutant, after 6 days at 29°C stained with Coracle (purple), FasIII (green) and Spectrin (green), showing defective cyst packaging and incomplete separation of egg chambers. Arrow marks enlarged egg chamber containing multiple cysts. Arrowhead marks two successive egg chambers lacking intervening stalk. D) Ovariole of N ts1 mutant restored to 18°C for 9 days after 6 days at 29 °C. Egg chamber formation is restored back to wild type morphology. [file 12861_2015_59_MOESM2_ESM.jpeg]

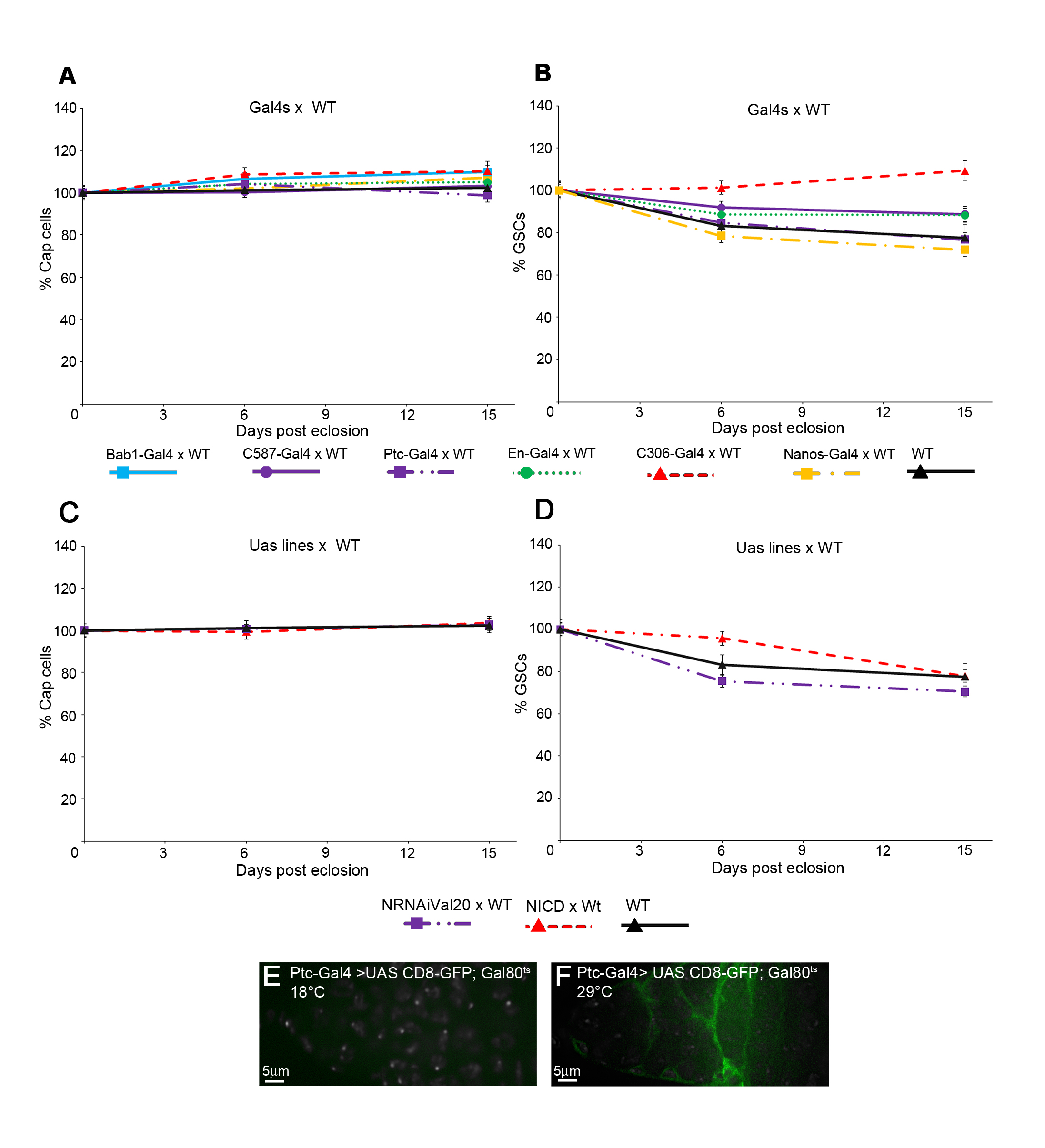

Supplement: Additional file 3: Figure S3. — Cap cell and GSC scoring of Gal4 and UAS lines. (A-D) Scoring of cap cell (A, C) and GSC numbers (B, D) over time since eclosion for Gal4 lines (A, B) and UAS lines (C, D) used, after out-crossing to WT. Data shown as mean ± SEM and normalised to % of cap cell and GSC numbers on eclosion (n = 37-60). (E, F) temperature-dependent Gal4-dependent expression demonstrated using UAS-CD8-GFP driven by PtcGal4 coexpressed with Gal80ts. No GFP detected at 18°C (E) but clear expression observed at 29°C (F). [file 12861_2015_59_MOESM3_ESM.jpeg]
